# Rhizosphere Competence and Biocontrol Effect of *Pseudomonas* sp. RU47 Independent from Plant Species and Soil Type at the Field Scale

**DOI:** 10.3389/fmicb.2018.00097

**Published:** 2018-02-01

**Authors:** Susanne Schreiter, Doreen Babin, Kornelia Smalla, Rita Grosch

**Affiliations:** ^1^Institute for Epidemiology and Pathogen Diagnostics, Federal Research Centre for Cultivated Plants, Julius Kühn-Institut (JKI), Braunschweig, Germany; ^2^Department Plant-Microbe Systems, Leibniz Institute of Vegetable and Ornamental Crops, Großbeeren, Germany

**Keywords:** *Rhizoctonia solani*, plant health, biocontrol, plant disease, bacterial community, DGGE

## Abstract

Biocontrol inoculants often show inconsistency in their efficacy at field scale and the reason for this remains often unclear. A high rhizosphere competence of inoculant strains is assumed to be a key factor for successful biocontrol effects as the biocontrol strain has to compete with the indigenous microbial community in the rhizosphere. It is known that many factors, among them plant species and soil type shape the rhizosphere microbial community composition. However, microbial community composition in the rhizosphere can also be influenced by the presence of a pathogen. We hypothesized that plant species, soil type, and a pathogen affect the rhizosphere competence of a biocontrol strain and its biocontrol effect against a soil-borne pathogen. To test the hypothesis, we used an experimental plot system with three soil types (diluvial sand, alluvial loam, loess loam) kept under similar agricultural management at the same field site for 12 years. We investigate the rhizosphere competence of *Pseudomonas* sp. RU47 in two plant species (potato and lettuce) and its biocontrol effect against Rhizoctonia diseases. The colonization density of a rifampicin resistant mutant of RU47 in the rhizosphere of both crops was evaluated by plate counts. Bacterial community compositions were analyzed by denaturing gradient gel electrophoresis (DGGE) of 16S rRNA gene fragments amplified from total community DNA. The inoculant RU47 was able to colonize the rhizosphere of both model crops in a sufficient density and to reduce disease severity of black scurf on potato and bottom rot on lettuce in all three soils. DGGE indicated that RU47 affected the bacterial community composition stronger in the rhizosphere of lettuce than in the potato rhizosphere. In contrast, the effect of the pathogen *Rhizoctonia solani* on the bacterial community was much stronger in the rhizosphere of potato than in the lettuce rhizosphere. A significant effect of RU47 on the *Pseudomonas*-specific *gacA* fingerprints of the rhizosphere was only observed in lettuce in alluvial soil. The soil type and plant species independent biocontrol effects of RU47 and its minor influence on the indigenous bacterial community composition might be important criteria for the registration and use of RU47 as biocontrol strain.

## Introduction

Diseases caused by soil-borne pathogens such as *Rhizoctonia solani* (Kühn) are difficult to control, and the use of bacterial inoculants for disease suppression represents an environmentally friendly control method (Alabouvette et al., 2009). Still biocontrol inoculants often showed a lack of consistency in their biocontrol activity at the field scale (Barret et al., 2011).

Over decades, efforts have been made to unravel factors affecting biocontrol efficacy of bacterial inoculants (Bais et al., 2006; Barret et al., 2011; Ghirardi et al., 2012). Variation in the ability of bacterial inoculants to colonize the rhizosphere and survive at sufficiently high cell densities was thought to be a reason for inconsistency in disease suppression (Ghirardi et al., 2012). Therefore, a high rhizosphere competence was identified as a prerequisite for the expression of beneficial effects on plants (De Bellis and Ercolani, 2001; Barret et al., 2011; Ghirardi et al., 2012).

Research presently is just starting to unravel the complex interplay between plants and their microbiome including bacterial inoculants applied (Berendsen et al., 2012; van der Heijden and Hartmann, 2016; Berg et al., 2017). Studies done by 16S rRNA gene-based fingerprints provided a growing body of evidence that biotic factors (e.g., plant species/genotype, developmental stage, and plant pathogens) and abiotic factors (e.g., weather conditions, agricultural management) affect the bacterial community composition in the rhizosphere (Berg and Smalla, 2009; Weinert et al., 2009; Schreiter et al., 2014a).

Several studies have clearly shown that both plant and soil type shape the rhizosphere microbiome (Bulgarelli et al., 2012; Peiffer et al., 2013; Schreiter et al., 2014b). Different soil types varying in physicochemical properties were shown to contain distinct bacterial communities from which a subset of populations was enriched in the rhizosphere of the plant due to root exudates (Costa et al., 2006; Schreiter et al., 2014b) resulting in an increased relative abundance in the rhizosphere compared to the corresponding bulk soil.

Until recently, limited information was available on the effect of different soil types and the plant species on the rhizosphere competence and biocontrol activity of bacterial inoculants at field scale. Results of a previous study showed that although the soil types harbor a specific bacterial community no impact on the ability of the inoculant strains *Serratia plymuthica* 3Re4-18 and *Pseudomonas* sp. RU47 to colonize the rhizosphere of lettuce in a sufficient density was given at the field scale (Schreiter et al., 2014c).

The strain RU47, initially identified as *Pseudomonas jessenii* based on the 16S rRNA gene sequence, has been recently reclassified as its genome sequence is phylogenetically related to strains from *P. koreensis* group (Eltlbany et al., under revision). Based on the results of this study, we assume that the often-reported inconsistency of biocontrol effects in the field is more likely due to plant characteristics. The plant modulates via root exudates the rhizosphere microbiome by stimulating microorganisms with traits beneficial for plant growth and health (Dennis et al., 2010; Doornbos et al., 2012). About 30–50% of photosynthetically fixed carbon are released through the root system in the rhizosphere (Kumar et al., 2006). Hence, root exudates play an important role in provoking a substrate-driven competition between rhizosphere microorganisms including an applied biocontrol inoculant in colonization of roots (Bais et al., 2006). The root exudation pattern and the amounts of various exudate compounds synthesized and exuded by the roots are under the plant’s genetic control (Micallef et al., 2009). Therefore, the plant species and its unique root exudation pattern attract a specific rhizomicrobial community (Chapparo et al., 2014). A biocontrol inoculant introduced into the rhizosphere has to compete and interact with this plant specific microbial community. Moreover, the rhizosphere is a highly dynamic habitat due to the changing input by the plant. As a result, the rhizosphere microbial community composition and activity differs temporally and spatially (DeAngelis et al., 2009; Chowdhury et al., 2013).

The main objective of our study was to assess the rhizosphere competence and biocontrol activity of the bacterial inoculant RU47 against a soil-borne pathogen depending on the plant species in three soil types.

In 2011 the ability of RU47 to colonize the rhizosphere of lettuce and its biocontrol of *R. solani* was shown to be soil type independent but strikingly dependent on plant growth (Schreiter et al., 2014c). In the present study we compare the rhizosphere competence and biocontrol effects of the inoculant RU47 on lettuce (*Lactuca sativa* L.) with its rhizosphere competence and biocontrol effects on potato (*Solanum tuberosum* L.). There are great differences in systematic botany, phenotype and cultivation period between the model plants. The used model plant species potato and lettuce are members of different plant families (*Solanaceae* and *Asteraceae*, respectively) and the growing period for lettuce is around 5–6 weeks in the field, compared to potato with a cultivation period of around 4–5 months. We expect a pronounced different bacterial community structure in the rhizosphere of the used model plants. Both potato and lettuce are host plants of the soil-borne pathogen *R. solani*.

It is important to note that the fungal pathogen *R. solani* is a species complex of various genetic groups called anastomosis groups (AGs) with a distinct degree of host specificity. While *R. solani* AG1-IB is responsible for bottom rot on lettuce, *R. solani* AG3 causes black scurf disease on potato with striking differences in the genome (Wibberg et al., 2013) and in the interaction with the plant. Whereas *R. solani* AG3 colonizes the belowground parts of potato including the roots (Genzel et al., 2018), *R. solani* AG1-IB infects lettuce via the lower leaves in contact to the soil (Grosch et al., 2011). Hence, we expect a stronger impact of *R. solani* AG3 on the microbial community in the rhizosphere of potato than of *R. solani* AG1-IB in the rhizosphere of lettuce.

In view of the differences in plant-pathogen interaction of the two model plants we aimed to answer the question of whether RU47 is able to compete successfully with the bacterial community in the rhizosphere in both plant species independent from the soil type and to express biocontrol effects in the field on both crops. We used an experimental plot system with three soil types (diluvial sand, alluvial loam, loess loam) kept under similar agricultural management at the same field site. The effects of the inoculant strain RU47 as well as the pathogens on bacterial community composition in the rhizosphere were assessed by denaturing gradient gel electrophoresis (DGGE) analysis of 16S rRNA gene fragments amplified from total community (TC)-DNA. The synthesis of secondary metabolites involved in antagonistic interactions and disease suppression is positively controlled by the global response regulator gene *gacA* (Haas and Defago, 2005). Being highly conserved among *Pseudomonas* species, *gacA* serves as a reliable phylogenetic marker for community fingerprinting (De Souza et al., 2003). Hence, we used *gacA* in addition to the 16S rRNA gene to analyze the effect of RU47 and the pathogens *R. solani* AG3 and AG1-IB on the bacterial and the *Pseudomonas* community in the rhizosphere of both model plants.

## Materials and Methods

### Field Experiments

A unique experimental plot system with independent experimental units for lettuce (unit 6) and potato (unit 7) was used for the experiments at the Leibniz-Institute of Vegetable and Ornamental Crops in Großbeeren (52° 33^′^ N, 13° 22^′^ E). Each unit consisted of three soil types characterized as Arenic-Luvisol (diluvial sand), Gleyic-Fluvisol (alluvial loam), and Luvic-Phaeozem (loess loam) that shared the comparable agricultural management at the same field site for more than 10 years (Rühlmann and Ruppel, 2005; Schreiter et al., 2014a). Each soil type was arranged in a separate block with 24 plots of 2 m × 2 m and a depth of 75 cm. From 2000 to 2011, following crops were cultivated in unit 6: pumpkin, nasturtium, pumpkin, amaranth, wheat, wheat, pumpkin, nasturtium, wheat, wheat, lettuce and lettuce; and in unit 7: broccoli, phacelia, broccoli, nasturtium, nasturtium, tomato, soybean, phacelia, wheat, phacelia, nasturtium, and phacelia.

On 5 May 2012, seed potato tubers (cv. Arkula, Norika GmbH, Groß Lüsewitz, Germany) were planted in unit 7 at a distance of 30 cm within a row, and with an intra-row distance of 65 cm. The number of tubers (diameter 35–60 mm) and the marketable tuber yield (MTY) were assessed at harvest 18 weeks after planting (on 5 September 2012). Additionally, the percentage of infestation of 20 randomly selected tubers per replicate with *Rhizoctonia* sclerotia was evaluated on a scale from 1 to 5 (1 – without sclerotia, 2 – <1% infestation, 3 – 5% infestation, 4 – 10% infestation, 5 – ≥15% infestation).

Seedling trays filled with the respective soil type were used to cultivate the lettuce seedlings (cv. Tizian, Syngenta, Bad Salzuflen, Germany) as described by Schreiter et al. (2014c). Lettuce seedlings were planted on 3 July 2012 at the three- to four-leaf stage 4 weeks after sowing with a within-row and intra-row distance of 30 cm to the experimental plot unit 6. The lettuce shoot dry mass (SDM) of each plant and the disease severity of bottom rot were assessed according to the following scale: (1) healthy plants without bottom rot symptoms; (2) symptoms on first lower leaves and small brown spots on the underside of leaf midribs; (3) brown spots on leaf midribs on lower and next upper leaf layers; and (4) severe disease symptoms on upper leaf layers and beginning of head rot to total head rot (Supplementary Figure S1) at harvest five weeks after planting (on August 8, 2012; typical size, form and firmness of head reached).

In both experimental units, plots of each soil type were adjusted to the same nitrogen amount of 157 kg ha^-1^ (Kalkamon, 27% N) before planting, based on nutrient analysis of the soil which was done according to the certified protocols of the Association of German Agricultural Analytic and Research Institutes (VDLUFA). The soils in unit 7 (potato) were also adjusted to the same amount of potassium of 210 kg ha^-1^ (Patentkali, K+S Kali GmbH, Kassel, Germany). Both crops were overhead irrigated based on the computer program ‘BEREST.’

The following treatments of potato and lettuce were studied: treatment without *Pseudomonas* sp. RU47 and without pathogen application (control), treatment with *R. solani* inoculation (+*Rs*), treatment with RU47 but without pathogen inoculation (RU47), and treatment with RU47 and *R. solani* inoculation (RU47+*Rs*). Each treatment included four replicates (plots) with 21 (potato) or 36 (lettuce) plants per replicate, randomly arranged per soil type.

### Inoculation of the Pathogens

The *R. solani* isolate Ben3 was obtained from sclerotia of mature potato tuber and characterized by molecular tools as AG3 (Kuninaga et al., 2000). The isolate *R. solani* AG1-IB 7/3/14 (AJ868459) was originally isolated from lettuce plants with bottom rot symptoms and characterized by conventional and molecular tools (Grosch et al., 2007). The inocula of both pathogens were prepared on barley kernels as described by Schneider et al. (1997).

For pathogen inoculation of potato at planting, seed tubers were covered with a 3 cm thick soil layer upon which five barley kernels infected with the black scurf pathogen *R. solani* AG3 were placed in plots with pathogen inoculation. Non-infected kernels were placed on tubers in plots without pathogen inoculation.

To ensure a homogeneous pathogen pressure for bottom rot in unit 6, a total of 36 lettuce plants (nine or more true leaves unfolded) were shredded and evenly incorporated in the top soil (10 cm) together with 40 g of barley kernels infected with *R. solani* AG1-IB or of non-infected barley kernels (plots without pathogen inoculation) with a rotary hoe 2 weeks before initiating the experiment.

### Preparation of RU47 Cell Suspension and Application Mode

A rifampicin resistant mutant of *Pseudomonas* sp.RU47 (strain collection of the Julius Kühn-Institut) was used to assess the colonization density in the field (Adesina et al., 2009). The inoculum of RU47 was prepared on King’s B agar plates (Merck KGaA, Darmstadt, Germany) for seed treatment, and in nutrient broth (NB II, SIFIN GmbH, Berlin, Germany) for treatment of young lettuce plants and seed potato tubers as described by Schreiter et al. (2014c). Both media were supplemented with rifampicin (75 μg mL^-1^). After a cultivation time of 16 h the culture was centrifuged at 13,000 *g* for 5 min, the supernatant discarded and the pellet was re-suspended in sterile 0.3% NaCl solution.

The potato seed tubers were inoculated with RU47 immediately before planting, spraying 20 mL suspension (10^8^ colony forming units [CFU] mL^-1^) on 10 tubers, and the emerged potato plants were inoculated four weeks after planting (WAP) by watering each plant with 100 mL suspension (10^8^ CFU mL^-1^).

A total of 100 lettuce seeds were coated on a Vortex Mixer (MSI, Minishaker, Staufen, Germany) with 250 μl of the bacterial cell suspension dropped on the seeds [10^8^ CFU mL^-1^]. Each lettuce seedling at its three-leaf stage was treated with 20 mL cell suspension of RU47 (10^7^ CFU mL^-1^) 1 week before transplanting them to the field plots and with 30 mL (10^8^ CFU mL^-1^) 2 days after planting. Regarding respective control treatments for lettuce and potato, watering was carried out with 0.3% NaCl.

### Sampling, Sample Processing and Determination of RU47 Counts

The rhizosphere competence of *Pseudomonas* sp. RU47 was analyzed on potato and on lettuce at two time points (7 and 12 WAP for potato, 2 and 5 WAP for lettuce) and the bacterial and *Pseudomonas* community composition (based on 16S rRNA or *gacA* gene, respectively) at one time point (7 WAP for potato, 2 WAP for lettuce) during the growth period of both plants.

Potato plants were sampled 7 WAP of seed potato tuber at growth stage 2 (formation of basal side shoots below and above soil surface) and 12 WAP at growth stage 7 (50% of berries in the first fructification have reached full size). The roots of two potato plants per replicate were combined at the first sampling time (7 WAP), and the roots of one plant per replicate were sampled at the second sampling time (12 WAP).

Lettuce plants were sampled 2 WAP when plants had nine or more true leaves unfolded, and 5 WAP when lettuce head reached typical size, form and firmness. For each treatment the roots of three lettuce plants per replicate were combined as a composite sample and considered as one replicate. In both crops the rhizosphere competence of RU47 and the rhizosphere bacteria of four replicates per treatment were analyzed.

Adhering soil was removed by a quick root wash step, and the root system was cut into 1 cm long pieces with sterile scissors and mixed before microorganisms were extracted as follows: 5 g of roots were placed in sterile Stomacher bags and treated by a Stomacher 400 Circulator (Seward Ltd., Worthing, United Kingdom) for 30 s at high speed after adding 15 ml of sterile 0.3% NaCl. The Stomacher blending step was repeated three times and one ml was taken from the combined 45 ml for plate counts while the microbial rhizosphere/rhizoplane fraction was harvested by centrifugation steps as described by Schreiter et al. (2014a).

Stomacher blending steps were immediately processed to determine the CFU of RU47 by plating serial dilutions on King’s B agar supplemented with rifampicin (75 μg mL^-1^) and cycloheximide (100 μg mL^-1^) after an incubation time of 48 h at 28°C. The CFU were calculated per gram root dry mass. For both plant species and all soil types, Stomacher supernatants obtained from the control plots were plated as well to determine the background of the indigenous rifampicin resistant bacteria.

### Total DNA Extraction and Analysis of 16S rRNA and *gacA* Gene Fragments by DGGE Fingerprints

Total community DNA was extracted after a harsh lysis step by means of the FastDNA SPIN Kit for Soil^®^ (MP Biomedicals, Heidelberg, Germany) from the same samples used for RU47 CFU determination (Schreiter et al., 2014a). PCR reactions were performed for amplification of 16S rRNA gene fragments with the bacterial primers F984-GC and R1378 using GoTaq^®^ Flexi (Promega, Mannheim, Germany) (Nübel et al., 1996; Heuer et al., 1997). The PCR products were analyzed by DGGE as described by Weinert et al. (2009). For characterization of the effect of RU47 with (RU47+*Rs*) and without (RU47) *R. solani* inoculation on *Pseudomonas* community in the potato and lettuce rhizosphere, DGGE fingerprints of *gacA* gene amplicons were carried out according to Costa et al. (2007).

### Data Analysis

All data obtained from the field were analyzed with the STATISTICA program (StatSoft Inc., Tulsa, OK, United States). The impact of the soil type, of the inoculant RU47 and of the pathogen *R. solani* on MTY of potato and SDM of lettuce was analyzed using three-way ANOVA combined with Tukey *post hoc* test (*P* ≤ 0.05). Effects of RU47 and the pathogen on MTY and SDM were analyzed for each soil type by two-way ANOVA. The data of disease severity was analyzed using the non-parametric Kruskal–Wallis test followed by Mann–Whitney *U*-test (*P* ≤ 0.05). The CFU per gram root dry mass (RDM) were calculated and logarithmically (Log_10_) converted. The effects of the soil type, the growth period/plant age (sampling time), and the pathogen on the plate counts of RU47 were evaluated using three-way ANOVA (*P* ≤ 0.05) combined with Tukey *post hoc* test (*P* ≤ 0.05). DGGE fingerprints were evaluated with GELCOMPAR II version 6.5 (Applied Maths, Sint-Martens-Latem, Belgium) as described by Schreiter et al. (2014a). The Pearson correlation coefficient as a curve-based method was chosen to obtain pairwise similarities between fingerprints and similarities were clustered using unweighted pair group method using average linkages (UPGMA). These were used for statistical analysis by Permutation tests (*P* ≤ 0.05) where the *d*-value was calculated as average overall correlation coefficients within the groups minus the average overall correlation coefficients between samples from different groups as suggested by Kropf et al. (2004).

## Results

### Plant Species Effect on Rhizosphere Competence of RU47 in Three Soils

**FIGURE 1:**
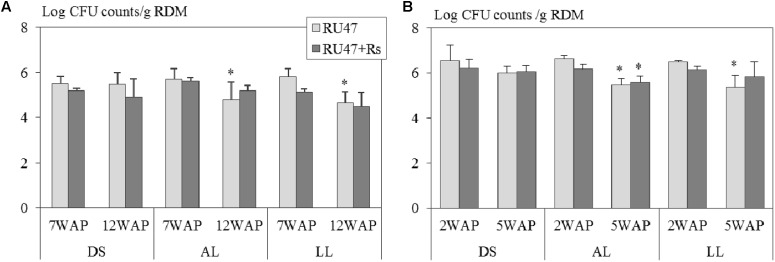
Number of colony forming units (CFU) per gram root dry mass (RDM) of *Pseudomonas* sp. RU47 estimated in the rhizosphere of **(A)** potato (cv. Arkula) and **(B)** lettuce (cv. Tizian) with RU47 and with RU47 + *Rhizoctonia solani* inoculation (RU47+*Rs*) 7 and 12 weeks after planting (7 WAP, 12 WAP) of potato and 2 and 5 weeks after planting (2 WAP, 5 WAP) of lettuce in the 2012 season. Plants were grown in three soils [diluvial sand (DS), alluvial loam (AL), loess loam (LL)] at the same field site. No significant effect of the soil type and the presence of *R. solani* on the number of CFU of RU47 was revealed (ANOVA, *P* ≥ 0.05). Asterisks indicate significant differences in CFU counts between the two sampling times within each soil type (Tukey *post hoc* test, *P* ≤ 0.05). The bars represent the standard deviation.

Our results demonstrated that *Pseudomonas* sp. RU47 was able to colonize the rhizosphere of potato and lettuce at a density of more than 5 Log_10_ CFU per gram root dry mass in all three soils at field scale at both sampling times. Three-way ANOVA revealed that neither the soil type (potato: *P* ≤ 0.19; lettuce: *P* ≤ 0.08) nor the presence of *R. solani* (AG3: *P* ≤ 0.10; AG1-IB: *P* ≤ 0.44) significantly affected the number of RU47 CFU counts in the rhizosphere of both crops. Two-way ANOVA showed for both crops significantly declined CFU counts of RU47 in the rhizosphere with increasing plant age in the treatments without (RU47) and with *R. solani* inoculation (RU47+*Rs*) in both loamy soils (alluvial loam: *P* ≤ 0.02; loess loam: *P* ≤ 0.002) but not in the diluvial soil (*P* ≥ 0.15). Interestingly, no plant age-dependent decline in the density of RU47 was observed in the rhizosphere of both crops in the treatments with *R. solani* inoculation (RU47+*Rs*) in all soils, except for lettuce grown in alluvial soil (**Figure 1**).

### Soil Type, *R. solani,* and of RU47 Effects on Yield of Potato and Lettuce

A significant effect of the soil type (*P* ≤ 0.0001) and the pathogen *R. solani* AG3 (*P* ≤ 0.0001) on MTY was found but no effect of RU47 (*P* ≥ 0.15). Comparing the MTY of non-inoculated potato plants (control) in all soils, the lowest yield was recorded in diluvial sand (543.8 g plant^-1^) which differed significantly from MTY in both loamy soils (alluvial loam, 841.3 g plant^-1^; loess loam, 764.0 g plant^-1^; *P* ≤ 0.008) (**Table 1**). Two-way ANOVA indicated that the pathogen *R. solani* AG3 significantly reduced MTY of potato in both loamy soils (*P* ≤ 0.02) but not in diluvial sand (*P* ≤ 0.18) compared with control plants, respectively. In all soils, no effect by the inoculant RU47 on MTY of potato was observed in comparison to the non-inoculated control plants in absence (control vs. RU47) and in presence of *R. solani* (+*Rs* vs. RU47+*Rs*).

**Table 1:** Marketable tuber yield (MTY) of potato (Po, cv. Arkula) and shoot dry mass (SDM) of lettuce (Le, cv. Tizian) and disease severity of black scurf on potato and bottom rot on lettuce without (control) and with *Pseudomonas* sp. RU47 (RU47), and without and with *Rhizoctonia solani* inoculation (+*Rs*; RU47+*Rs*) in the 2012 season.

		SDM or MTY [g/plant]				Disease severity			
	Soil	Control	RU47	+*Rs*	RU47+*Rs*	Control	RU47	+*Rs*	RU47+*Rs*
	DS	543.8 aA	582.3 a	499.9 aA	516.8 a	1.1 aA	1.2 a	3.4 aA^∗^	2.2 b
Po	AL	841.3 aB	737.4 a	705.6 aB^∗^	643.2 a	1.1 aA	1.0 a	2.8 aB^∗^	1.7 b
	LL	764.0 aB	711.3 a	606.1 aAB^∗^	566.6 a	1.2 aA	1.1 a	2.8 aB^∗^	1.8 b
	DS	42.2 aA	39.8 a	31.9 aA^∗^	38.1 b	2.3 aA	2.4 a	2.8 aA^∗^	2.5 b
Le	AL	32.4 aB	32.0 a	25.5 aB^∗^	31.4 b	2.4 aA	2.2 a	3.0 aA^∗^	2.5 a
	LL	30.6 aB	30.9 a	25.3 aB^∗^	28.7 a	2.1 aA	2.1 a	2.6 aA^∗^	2.3 b

*Both crops were grown in three soils [diluvial sand (DS), alluvial loam (AL), loess loam (LL)] at the same field site. Asterisks denote significant differences in disease severity, SDM or MTY between control and pathogen control (+Rs), and different capital letters indicate significant differences comparing controls (control, +Rs; per column) of each soil type. Different small letters denote significant differences between treatments with RU47 and corresponding controls (control or +Rs) in each soil type (per row).*

Three-way ANOVA indicated a significant effect of the soil type (*P* ≤ 0.0001) and the pathogen (*P* ≤ 0.0001) but also not of RU47 (*P* ≥ 0.07) on lettuce SDM. In contrast to potato, the highest SDM of lettuce was recorded in diluvial sand (42.2 g plant^-1^) which also differed significantly from SDM in both loamy soils (alluvial loam, 32.4 g plant^-1^; loess loam, 30.6 g plant^-1^; *P* ≤ 0.0003). The SDM was significantly reduced by *R. solani* AG1-IB in all soils compared to the control plants (**Table 1**). The inoculant RU47 did not improve the SDM of lettuce in any of the soils in the treatments without *R. solani* inoculation (control vs. RU47; *P* ≥ 0.45), but it significantly enhanced the lettuce growth in diluvial sand (*P* ≤ 0.01) and alluvial loam (*P* ≤ 0.01) in treatments with *R. solani* inoculation (+*Rs* vs. RU47+*Rs*).

### Soil Type Effect on the RU47 Mediated Biocontrol Activity against *R. solani*

In all control treatments no, or only slight black scurf symptoms on potato tuber appeared at harvest. In contrast the inoculation of *R. solani* AG3 (+*Rs*) resulted in a significantly increased disease severity of black scurf on potato (*P* ≤ 0.05) in all soils (**Table 1**). Comparing disease severity of black scurf symptoms on potato in the pathogen controls (+*Rs*) of all three soils the highest and significant (*P* ≤ 0.03) disease severity was recorded on tubers harvested from diluvial sand. Comparable severity of black scurf (*P* ≤ 0.89) was observed on tubers yielded from plants grown in alluvial loam and loess loam (**Table 1**). RU47 was able to significantly suppress (*P* ≤ 0.03) the severity of black scurf on potato in all soils (+*Rs* vs. RU47+*Rs*).

Bottom rot symptoms on lettuce were revealed in the controls in each soil (**Table 1**). A significantly higher disease severity of bottom rot was revealed on plants in all soils after *R. solani* AG1-IB (+*Rs*) inoculation (*P* ≤ 0.05), while no significant differences (*P* ≥ 0.06) in disease severity were observed among the three soil types without (control) and with pathogen inoculation (+*Rs*; **Table 1**). The inoculant RU47 suppressed significantly (*P* ≤ 0.03) the severity of bottom rot on lettuce in diluvial sand and loess loam (+*Rs* vs. RU47+*Rs*). The disease severity of bottom root on plants grown in alluvial soil was reduced through RU47 application but not significantly (*P* ≤ 0.1).

### *R. solani* and RU47 Effects on the Rhizosphere Bacterial Community Composition

Effects of the plant species in three soil types, the pathogens *R. solani* AG3 and AG1-1B, and inoculant RU47 on the bacterial communities were detected based on 16S rRNA gene DGGE fingerprints for samples taken 7 or 2 WAP of potato or lettuce plants, respectively.

DGGE fingerprints revealed distinct rhizosphere bacterial communities depending on the plant species (Supplementary Figures S2–S4). Significant effects of *R. solani* AG3 on rhizosphere bacterial communities of potato plants were observed with high *d-*values (**Table 2**), while the effects of AG1-1B on the bacterial community composition of the lettuce rhizosphere were negligible, indicated by low and not significant *d*-values (**Table 2**). The highest impact of both pathogens (AG3 and AG1-IB) on the rhizosphere bacterial community of potato and lettuce plants was revealed in alluvial loam (**Table 2**), indicating a soil type effect on the interaction of pathogen and indigenous bacterial community in the rhizosphere.

**Table 2:** *D*-values obtained by bacterial DGGE analysis of rhizosphere samples from potato (Po, cv. Arkula) and lettuce (Le, cv. Tizian) grown in three soils [diluvial sand (DS), alluvial loam (AL), loess loam (LL)] at the same field site.

		Effect of *Rs*			Effect of RU47			Effect of RU47+*Rs*		
	Soil	*d*-value	*p*-value	Figure	*d*-value	*p*-value	Figure	*d*-value	*p*-value	Figure
Po	DS	16.91^∗^	0.03	S2	6.23	0.09	S2	6.45^∗^	0.03	S2
	AL	22.08^∗^	0.03	S3	5.25	0.22	S3	16.7	0.06	S3
	LL	17.72^∗^	0.03	S4	0.75	0.28	S4	3.69	0.17	S4
Le	DS	0.73	0.23	S2	5.88^∗^	0.03	S2	7.83^∗^	0.03	S2
	AL	3.75	0.08	S3	19.7^∗^	0.03	S3	10.41^∗^	0.03	S3
	LL	3.02	0.06	S4	7.68^∗^	0.03	S4	4.38^∗^	0.03	S4

*D-values (in %) show the differences in the bacterial community composition between the control plots and plots treated with pathogen Rhizoctonia solani (Rs), biocontrol strain RU47, or RU47 and R. solani (RU47 + Rs). An asterisk indicates significant differences acquired by Permutation test as suggested by Kropf et al. (2004). A higher d-value indicates a more pronounced influence of the pathogen Rs, the inoculant strain RU47 or both RU47 + R. solani (RU47 + Rs).*

The inoculant RU47 showed plant species dependent effects on the rhizosphere bacterial community composition as well. Based on the DGGE fingerprints and the *d-*values obtained by the comparison of the control and the treatment with RU47 fingerprints, the effects of RU47 were on average lower and not significant in the rhizosphere of potato in contrast to the rhizosphere of lettuce (**Table 2**).

An effect on the lettuce rhizosphere bacterial community composition was observed in the treatment with RU47 in presence of the pathogen *R. solani* for both crops. The highest effect was found consistently in alluvial loam albeit not significant for potato (**Table 2**).

### RU47 and *R. solani* Effects on *Pseudomonas* Community in the Rhizosphere

The *Pseudomonas* community of the rhizosphere of both plant species was analyzed by DGGE of *gacA* genes for samples taken 7 or 2 WAP, respectively. A significant effect of the soil type was observed for lettuce in contrast to the potato rhizosphere which exhibited higher heterogeneity between replicates (control treatments in Supplementary Figures S5, S6). Inoculation with RU47 resulted only in small shifts of *Pseudomonas* community that were significant only for lettuce in alluvial loam soil (**Table 3**). The additional presence of *R. solani* AG3 (potato) or AG1-IB (lettuce) significantly changed the *Pseudomonas* community of potato grown in DS and LL and for lettuce grown in DS soil.

**Table 3:** *D*-values obtained by *Pseudomonas*-specific DGGE analysis of rhizosphere samples from potato (Po, cv. Arkula) and lettuce (Le, cv. Tizian) grown in three soils [diluvial sand (DS), alluvial loam (AL), loess loam (LL)] at the same field site.

		Effect of RU47			Effect of RU47+*Rs*		
	Soil	*d*-value	*p*-value	Figure	*d*-value	*p*-value	Figure
Po	DS	6.6	0.11	S6	7.5^∗^	0.04	S6
	AL	4.3	0.16	S6	5.3	0.16	S6
	LL	3.4	0.22	S6	8.3^∗^	0.03	S6
Le	DS	5.2	0.12	S5	11^∗^	0.03	S5
	AL	4.6^∗^	0.03	S5	6.4	0.06	S5
	LL	2.1	0.09	S5	0	0.62	S5

*D-values (in %) show the differences in the Pseudomonas community composition between the control plots and plots treated with biocontrol strain RU47 with or without inoculation of Rhizoctonia solani (RU47; RU47+Rs). An asterisk indicates significant differences acquired by Permutation test as suggested by Kropf et al. (2004). A higher d-value indicates a more pronounced influence of the pathogen R. solani or the inoculant strain RU47.*

## Discussion

In this study, we compared the ability of RU47 to colonize the rhizosphere and to display biocontrol effects against Rhizoctonia diseases for two plant species grown in three different soil types. Hereby we confirm results of previous years obtained with lettuce in the same long term field trial and broaden our knowledge with a systematic, morphological different model plant (Schreiter et al., 2014a,b,c). Both, potato and lettuce are host plants of *R. solani* but the interaction of the model plants vary with the different anastomosis groups (AGs) of *R. solani*. The model plants were grown in the same three soil types at the same site. However, they were planted in two units of the experimental plot system which exhibited only a slight variation in cropping history. In contrast to other field studies the experimental set-up excluded site effects caused by different weather conditions and management practice on bacterial communities in the bulk soil. Potato plants have a much longer growth period than lettuce, and thus the analysis of bacterial community was performed 2 and 7 WAP (3 and 8 weeks after last application of RU47). We assumed that the bacterial community structure in the rhizosphere differed depending on the plant species in each soil and pyrosequencing (Schreiter et al., unpublished) and DGGE analysis underlined this assumption (Supplementary Figures S2–S4).

Contrasting effects of the soil types on MTY of potato and the SDM of lettuce in the untreated controls were assessed at harvest. The soil characteristics play an important role in the ability of a plant species to extract water and nutrients. Although the soil types were adjusted to the same amount of nitrogen it cannot be excluded that soil nitrogen, i.e., nitrate, leaches by irrigation water to a deeper, non-rooted soil level, especially in the diluvial sand. Availability of nitrogen was possibly limited in potato crop in diluvial sand within the growth period of 12 weeks compared to the loamy soils.

A negative effect of the pathogen *R. solani* AG1-IB on lettuce growth and of *R. solani* AG3 on the MTY of potato was observed in all soils. These results confirmed the observation for lettuce made in previous field experiments (Grosch et al., 2005; Schreiter et al., 2014c). The disease impact of the bottom rot pathogen was not revealed in the present study conducted in 2012 when plants were treated with RU47 confirming the biocontrol effects of RU47 observed in 2011 (Schreiter et al., 2014c). Our results are in accordance with findings for the inoculant strains *Bacillus amyloliquefaciens* FZB42 and *Serratia plymuthica* 3Re4-18 for lettuce (Grosch et al., 2005; Chowdhury et al., 2013). However, we found that the inoculant RU47 was not able to compensate the negative effect of the pathogen on MTY of potato. Compared to *R. solani* AG1-IB on lettuce which does not colonize the roots, *R. solani* AG3 was previously reported to affect all belowground parts of potato including the roots during the growth period (Atkinson et al., 2011; Genzel et al., 2018). Thus, it was not surprising that *R. solani* AG3 had a more pronounced effect on the composition of the bacterial community in the rhizosphere of potato as indicated by the higher *d-*values than *R. solani* AG1-IB on the rhizosphere bacterial community in lettuce. Windisch et al. (2017) showed that both the pathogen and the biocontrol strain induced changes in the root exudation pattern of lettuce, including higher amounts of antimicrobial compounds. An impact of pathogens such as *Verticillium* sp. and *Pythium* sp. on root exudation patterns of cotton and basil plants was reported by Bais et al. (2006) and Zhang et al. (2011). It is assumed that *R. solani* AG3 may also cause changes in root exudation patterns of potato and thus affect the interaction of RU47 with the plant. Although RU47 was found in a sufficient density in the rhizosphere of potato and was able to reduce severity of black scurf symptoms in all soils the potato yield losses were not compensated.

Our results revealed that despite the plant species dependent rhizosphere bacterial community composition observed (Supplementary Figures S2–S4), the inoculant RU47 was able to successfully colonize the rhizosphere of potato and lettuce. The rhizosphere competence of RU47 in potato and lettuce was not influenced by the soil type, as already revealed in a previous experiment (Schreiter et al., 2014c). Higher CFU counts of RU47 were found in the rhizosphere of lettuce in comparison to the potato rhizosphere. The finding might be due to the differences in sampling time after RU47 application (3 and 8 weeks after the last application for potato; 2 and 5 weeks after the last application for lettuce). Furthermore, the CFU counts were calculated per gram of root dry mass and not per cm^2^ root surface. In contrast to potato, the fine root system of lettuce, which is likely to provide a higher root surface than the potato root system, might be a reason for the observed higher CFU counts in lettuce. A decline in the population density of RU47 within the growth period was found for both crops and confirmed previous observations of RU47 and the inoculant strains 3Re4-18, and FZB42 (Chowdhury et al., 2013; Schreiter et al., 2014c). It is assumed that the decrease in CFU counts is likely linked to changes in root morphology as the number of fine roots decreased with the growing plant. In both crops no impact of *R. solani* AG3 or AG1-IB on the rhizosphere competence of RU47 was revealed. These results were consistent with the study of Chowdhury et al. (2013) where the rhizosphere competence of FZB42 was also not affected by the bottom rot pathogen *R. solani* AG1-IB in lettuce. Our results underline that the ability of the inoculant RU47 to colonize the rhizosphere of potato and lettuce plants in a sufficient density correlated with effective biocontrol activity in both crops and in all three soils.

Black scurf symptoms were observed only on potato tubers originating from treatments inoculated with *R. solani* AG3. In contrast, the bottom rot symptoms on lettuce were also detected in all control treatments and confirmed previous reports on the occurrence of *R. solani* AG1-IB in arable soils (Grosch et al., 2011). In accordance with the results of the 2011 conducted field experiment (Schreiter et al., 2014c), the lowest disease severity of bottom rot on lettuce (control, +*Rs*) was assessed in loess loam (+*Rs*). But the differences in disease severity between the soil types were not significant in the treatment with *R. solani* AG1-IB as determined previously. An impact of the soil type on bean hypocotyl rot severity caused by *R. solani* AG4 was also revealed by Nerey et al. (2010). In addition, Tamm et al. (2010) showed that the soil type is a major determinant for suppressiveness against soil-borne diseases. Several studies suggested that physicochemical properties of different soils have a strong effect on microbial community structure and function (reviewed in Berg and Smalla, 2009). In accordance, we found distinct rhizosphere bacterial communities in the different soil types with the best soil disease suppression in loess loam (Schreiter et al., 2014c). The disease severity of bottom rot in the growing period 2011 was much higher in all soils compared to the growing period 2012. Pathogen inoculum density affects the disease severity and additional inoculation increased the density in all soils. Moreover, results of previous experiments over different growing periods showed that also weather conditions have a pronounced impact on disease development (Grosch et al., 2011). In the growing period 2011 the conditions were more favorable for disease development of bottom rot compared to 2012.

Although DGGE fingerprints do not provide taxonomic information on community composition and responders to the treatments, they provide rapid insights into dynamic changes and the differences between treatments. Recently, we could show that the pyrosequencing data for lettuce rhizosphere and bulk soil from the experimental plot units confirmed the DGGE findings (Schreiter et al., 2014a,b).

The effect of RU47 on rhizosphere bacterial communities differed depending on plant species and soil type as revealed by bacterial and *Pseudomonas*-specific DGGE fingerprints. A significant effect of RU47 was found in all three soils in lettuce in the treatments without and with additional pathogen inoculation. This is in accordance to previous findings on lettuce where the impact of RU47 was assessed over three consecutive years. In the previous study an increasing effect of RU47 on rhizosphere bacterial communities could be observed over the years 2010 until 2012. Nevertheless, the influence of RU47 was negligible compared to community changes caused by the soil type (Schreiter et al., 2014b). The comparison of this and the previous study conducted in 2011 confirmed the results obtained for lettuce which therefore shows the importance of repeating field experiments for validation and reliable assessment of biocontrol agents. Furthermore, also a comparison of the impact of a biocontrol agent between economically important model plants is needed. Therefore, we used TC-DNA extracted from the same lettuce root samples for 2012 as used in Schreiter et al. (2014b), performed a separated amplification, DGGE run and analysis to compare to potato. In contrast to lettuce, on average a minor influence of RU47 on the total bacterial as well as *Pseudomonas* communities was revealed in the potato rhizosphere and might be explained by differences in the response of the plant-specific microbial community to RU47 inoculation. Recent results of Schreiter et al. (2014b) on lettuce in the 2011 field experiment indicated that different taxonomic groups responded to RU47 application depending on the soil type. An increased relative abundance of bacteria belonging to the genera *Bacillus* and *Paenibacillus* was observed in the rhizosphere of lettuce grown in alluvial loam. Thus, mutualistic effects of the inoculant with distinct enriched populations or indirect effects involving other bacterial rhizosphere populations cannot be excluded. Numerous strains of these genera were previously reported to display antagonistic activity and may support the observed biocontrol effect of RU47 (Kröber et al., 2014). The observed effect on rhizosphere bacterial communities in potato of the treatment RU47 in presence of the pathogen (RU47+*Rs*) seems to be caused by *R. solani* AG3. The strong effect of the pathogen on bacterial and *Pseudomonas* communities could not be compensated by the inoculant RU47.

## Conclusion

The results underline that the bacterial community structure in the rhizosphere differed depending on the plant species in all soils analyzed. Despite the influence of the plant, the soil type, and the pathogen, the inoculant RU47 was capable of colonizing the rhizosphere of both crops in a density sufficient manner to reduce black scurf disease severity on potato and bottom rot on lettuce in all three soils. In both crops the rhizosphere competence of RU47 was neither affected by the soil type nor by the presence of the respective pathogen *R. solani*. In contrast to lettuce, the colonization of the belowground part of potato including the roots by the pathogen *R. solani* AG3 has had a pronounced effect on the bacterial community composition in the potato rhizosphere. Interestingly, the pathogen *R. solani* affected the *Pseudomonas* community in both crops whereas the inoculant RU47 influenced especially the bacterial as well as the *Pseudomonas* community in the rhizosphere of lettuce. The plant species and soil type independent rhizosphere competence of RU47 and biocontrol of Rhizoctonia diseases observed under field conditions underlines the biocontrol potential of RU47.

## Author Contributions

KS and RG preparing the project proposal for third-party funds. KS, RG, and SS project planning, design and performance of experiments. SS and RG determination of rhizosphere competence and plant characteristics, disease assessment, analysis of these data. SS and DB molecular work and analysis of microbial community composition in the rhizosphere. SS, RG, KS, and DB preparation of the manuscript.

## Conflict of Interest Statement

The authors declare that the research was conducted in the absence of any commercial or financial relationships that could be construed as a potential conflict of interest.
