# Supplementary material for: Rhizosphere Competence and Biocontrol Effect of Pseudomonas sp. RU47 Independent from Plant Species and Soil Type at the Field Scale
Source: Front Microbiol. 2018 Feb 1;9:97. doi: 10.3389/fmicb.2018.00097 (PMC5799239; doi:10.3389/fmicb.2018.00097)
Supplement: Supplementary file 1 [file Image_1.PDF]

SUPPORTING MATERIALS

for the article

Rhizosphere competence and biocontrol effect of *Pseudomonas* sp. RU47 independent from plant species and soil type at the field scale

Susanne Schreiter[1#], Doreen Babin[1], Kornelia Smalla[1] and Rita Grosch[2]

[1]Julius Kühn-Institut, Federal Research Centre for Cultivated Plants (JKI), Institute for Epidemiology and Pathogen Diagnostics, Messeweg 11-12, 38104 Braunschweig, Germany
[2]Leibniz-Institute of Vegetable and Ornamental Crops (IGZ), Department Plant-microbe systems, Theodor-Echtermeyer-Weg 1, 14979 Großbeeren, Germany
[#]Present address: Department of Sustainable Agriculture Science, Rothamsted Research, AL5 2JQ, Harpenden, UK

*__Correspondence:__ R. Grosch, [2]Leibniz-Institute of Vegetable and Ornamental Crops Großbeeren/Erfurt e.V. (IGZ), Theodor-Echtermeyer Weg 1, 14979 Großbeeren, grosch@igzev.de

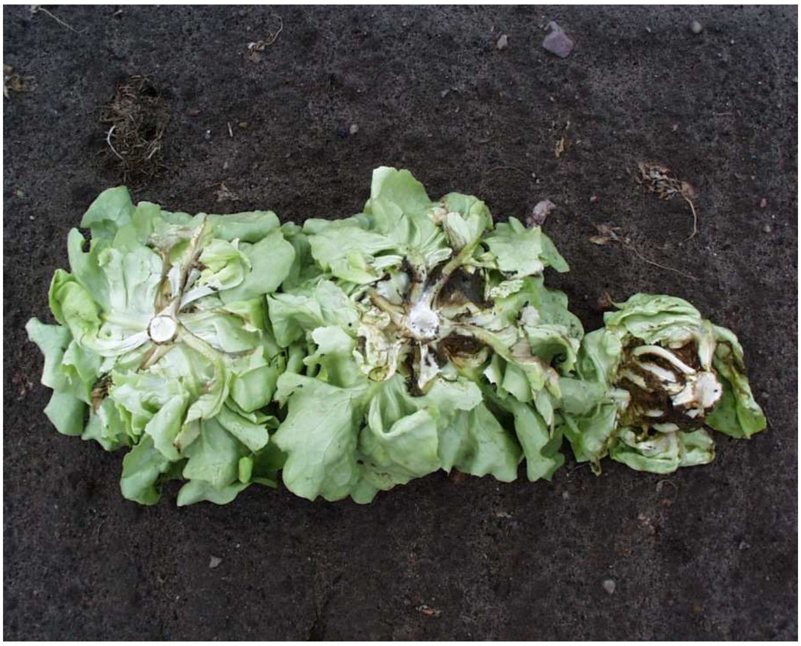

Fig. S1 Bottom rot symptoms on lettuce and disease severity scale: (from left to right) 2 – symptoms on first lower leaves and small brown spots on the underside of leaf midribs; 3 – brown spots on leaf midribs on lower and next upper leaf layers; 4 – severe disease symptoms on upper leaf layers and beginning of head rot.

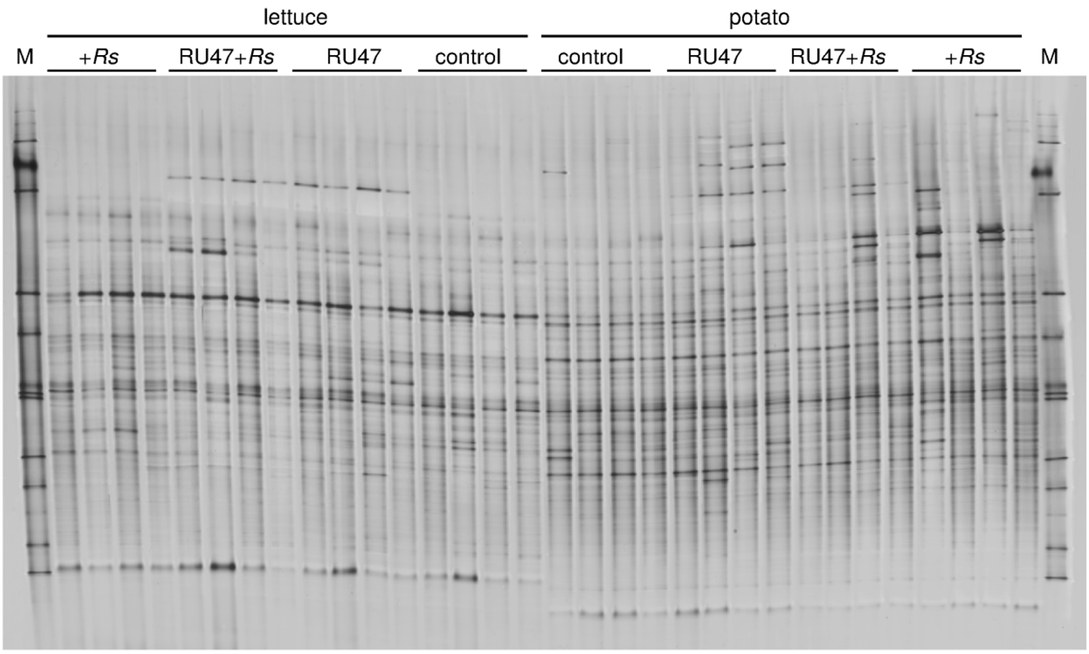

(A)

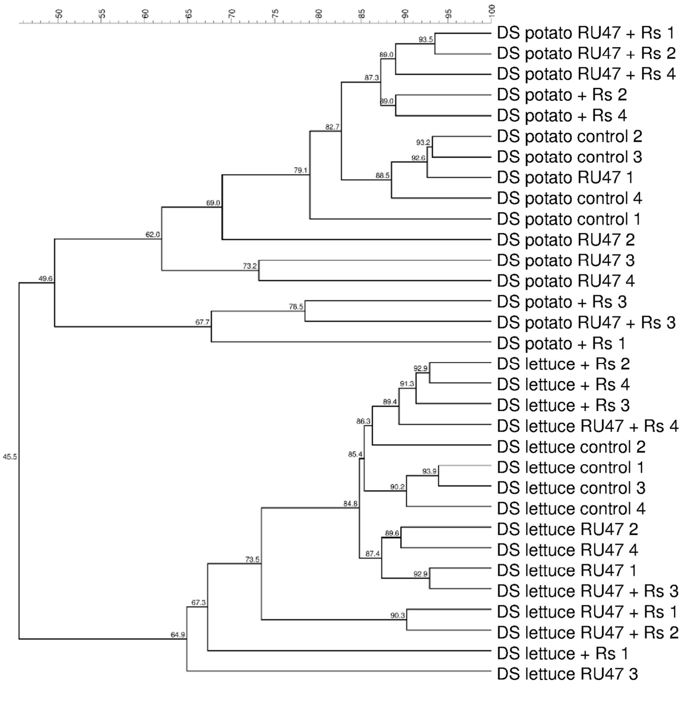

(B)

Fig. S2 DGGE fingerprints (A) of bacterial 16S rRNA gene fragments amplified from community DNA extracts. Samples from the control, *Pseudomonas* sp. RU47, *Rhizoctonia solani* (*Rs*) inoculated, and RU47 and *R. solani* inoculation (RU47+*Rs*) plots were obtained from the rhizosphere of lettuce (two weeks after planting) and potato (seven weeks after planting) grown in diluvial sand (DS). The corresponding UPGMA dendrogram (B) analysis is based on the Pearson similarity matrix. M: Marker

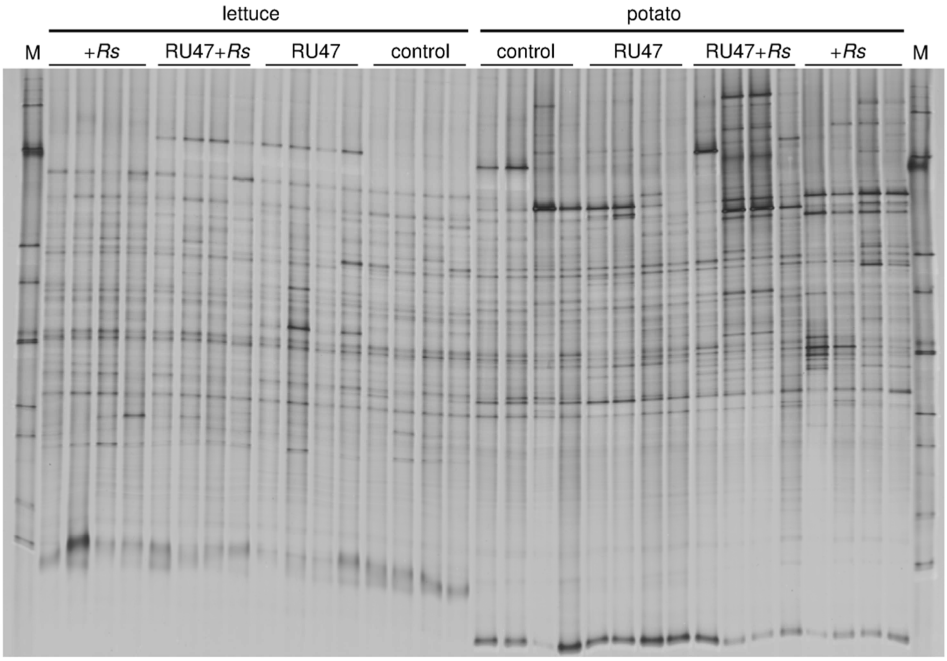

(A)

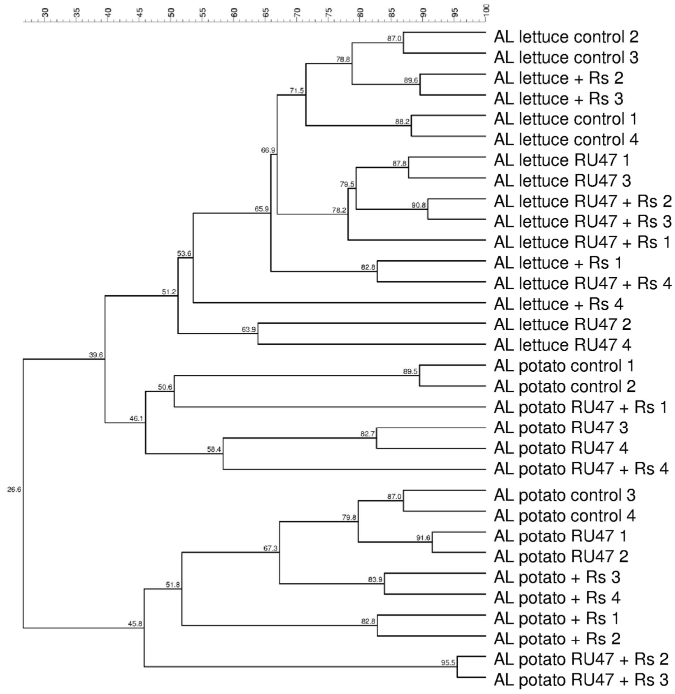

(B)

Fig. S3 DGGE fingerprints (A) of bacterial 16S rRNA gene fragments amplified from community DNA extracts. Samples from the control, *Pseudomonas* sp. RU47, *Rhizoctonia solani* (*Rs*) inoculated, and RU47 and *R. solani* inoculation (RU47+*Rs*) plots were obtained from the rhizosphere of lettuce (two weeks after planting) and potato (seven weeks after planting) grown in alluvial loam (AL). The corresponding UPGMA dendrogram (B) analysis is based on the Pearson similarity matrix. M: Marker

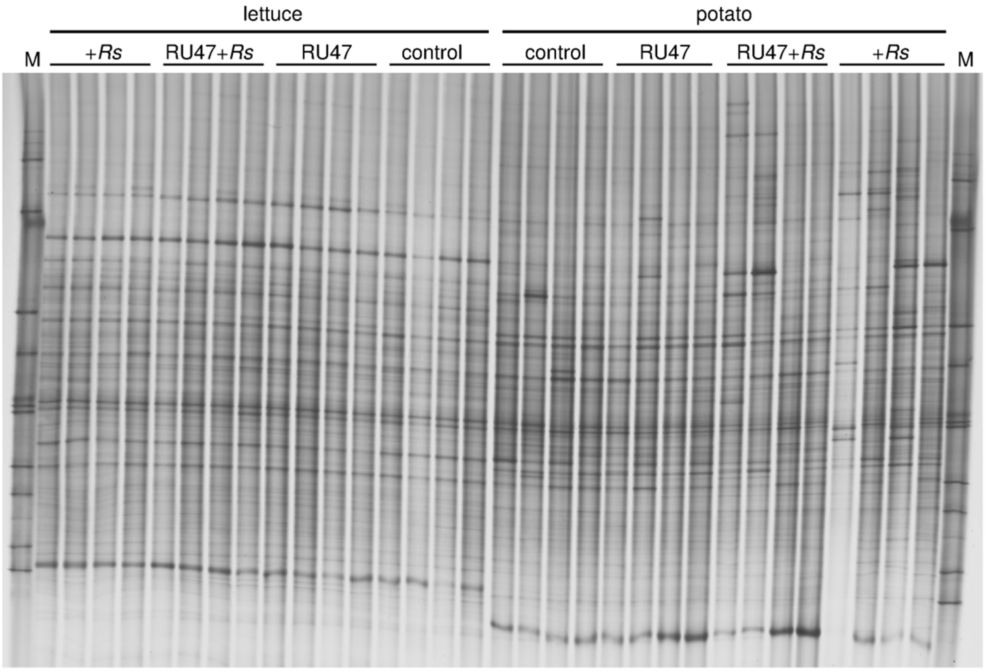

(A)

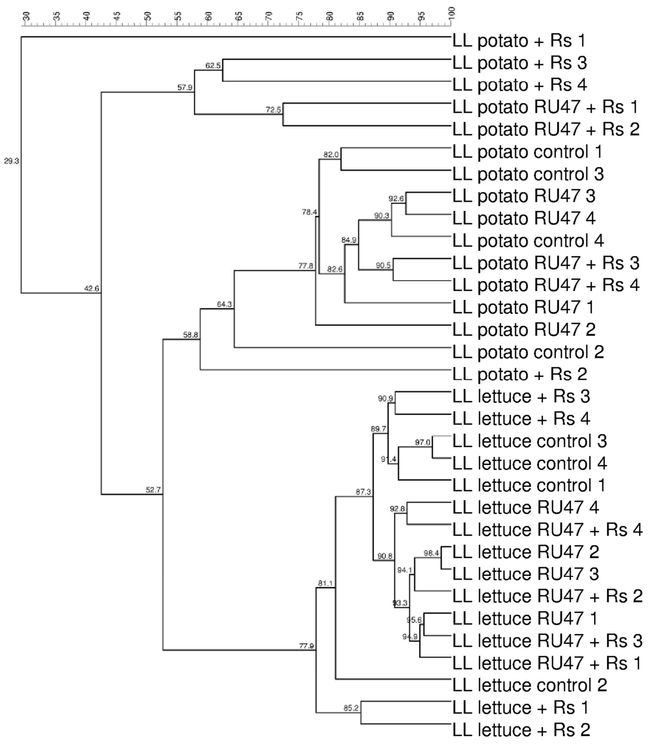

(B)

Fig. S4 DGGE fingerprints (A) of bacterial 16S rRNA gene fragments amplified from community DNA extracts. Samples from the control, *Pseudomonas* sp. RU47, *Rhizoctonia solani* (*Rs*) inoculated, and RU47 and *R. solani* inoculation (RU47+*Rs*) plots were obtained from the rhizosphere of lettuce (two weeks after planting) and potato (seven weeks after planting) grown in loess loam (LL). The corresponding UPGMA dendrogram (B) analysis is based on the Pearson similarity matrix. M: Marker

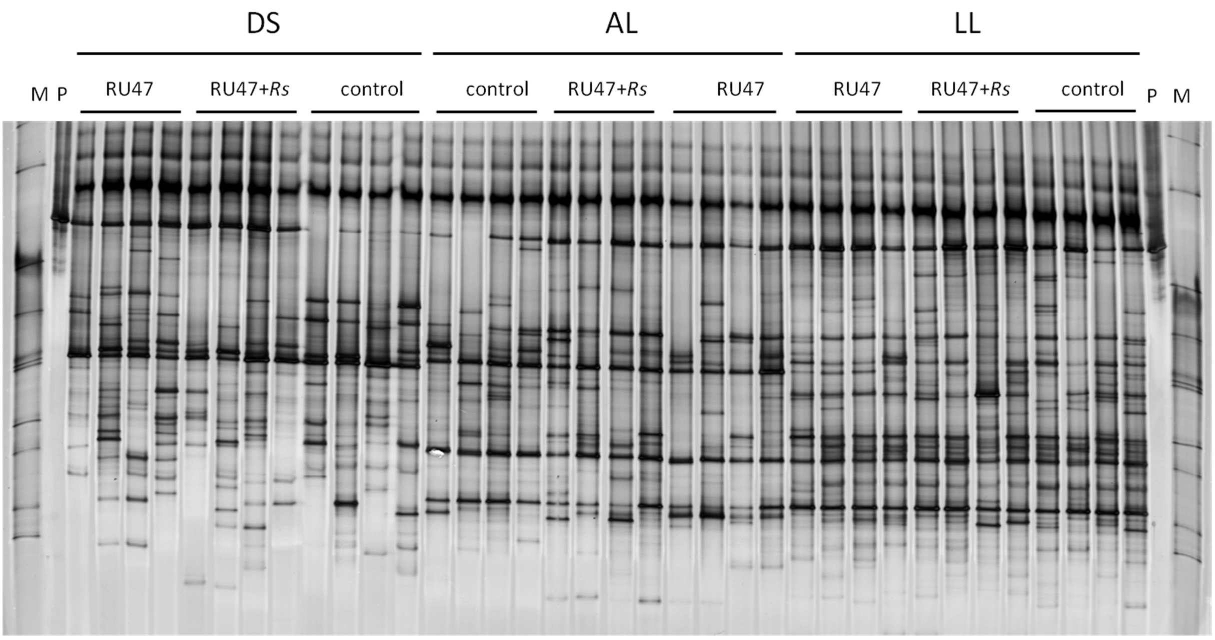

(A)

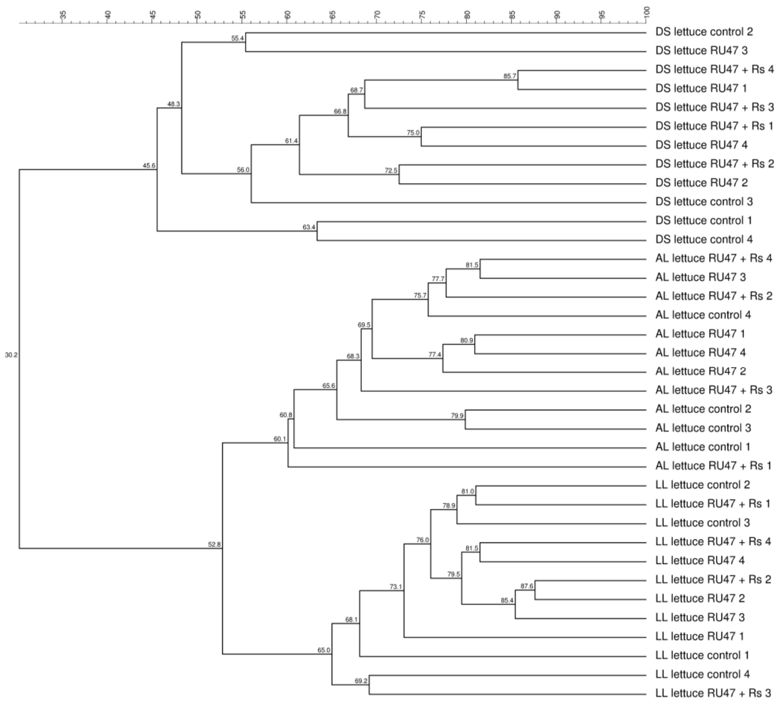

(B)

Fig. S5 DGGE fingerprints (A) of Pseudomonas-specific *gacA* genes amplified from community DNA extracts. Samples from the control, *Pseudomonas* sp. RU47, and RU47 and *Rhizoctonia solani* (*Rs*) inoculation (RU47+*Rs*) plots were obtained from the rhizosphere of lettuce (two weeks after planting) grown in three soils [diluvial sand (DS), alluvial loam (AL) and loess loam (LL)] at the same field site. The corresponding UPGMA dendrogram (B) analysis is based on the Pearson similarity matrix. M: Marker; P: positive control

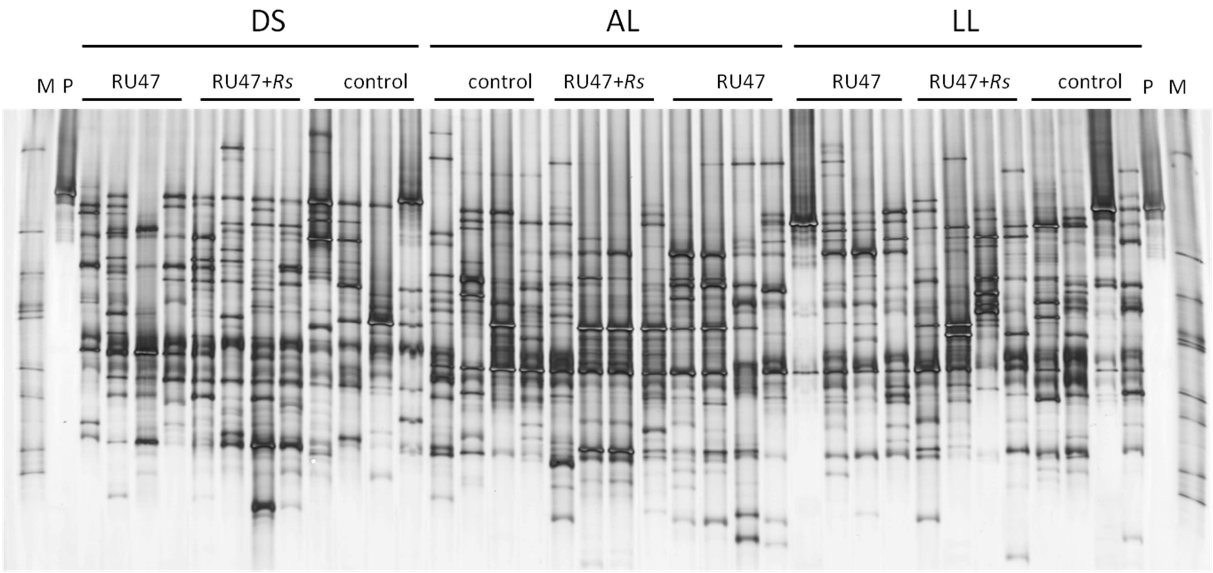

(A)

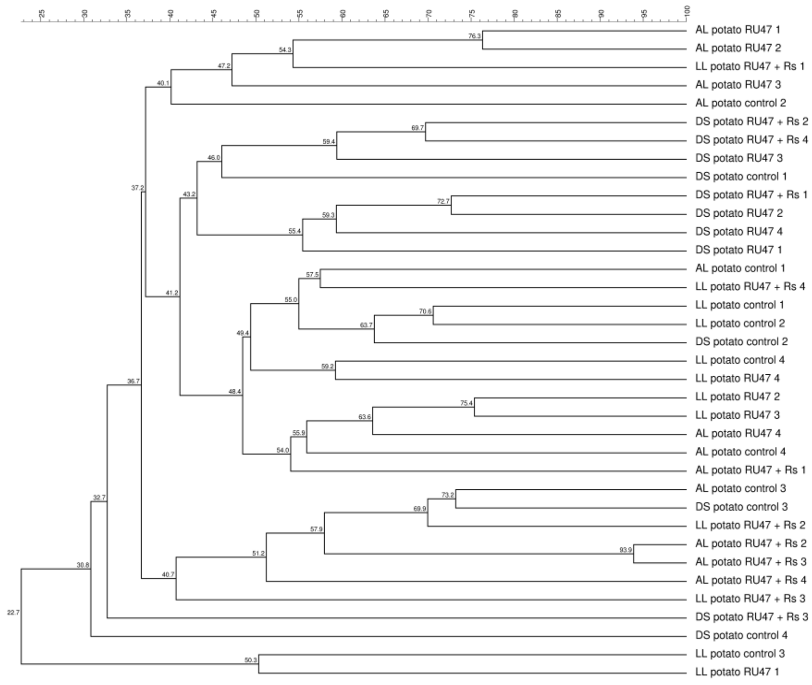

(B)

Fig. S6 DGGE fingerprints (A) of Pseudomonas-specific *gacA* genes amplified from community DNA extracts. Samples from the control, *Pseudomonas* sp. RU47, and RU47 and *Rhizoctonia solani* (*Rs*) inoculation (RU47+*Rs*) plots were obtained from the rhizosphere of potato (seven weeks after planting) grown in three soils [diluvial sand (DS), alluvial loam (AL) and loess loam (LL)] at the same field site. The corresponding UPGMA dendrogram (B) analysis is based on the Pearson similarity matrix. M: Marker; P: positive control
